# Emergence of Infective Endocarditis Due to *Serratia* spp.: Results of a Multicenter Cohort

**DOI:** 10.1093/ofid/ofaf036

**Published:** 2025-01-22

**Authors:** Leah Madeline McCrary, Douglas Slain, Sunish Shah, Bobbi Jo Stoner, Ashley H Marx, Asher J Schranz

**Affiliations:** Division of Infectious Diseases, Washington University in St. Louis School of Medicine, St. Louis, Missouri USA; Division of Infectious Diseases, University of North Carolina at Chapel Hill School of Medicine, Chapel Hill, North Carolina USA; Department of Clinical Pharmacy, West Virginia University, Morgantown, West Virginia, USA; Department of Pharmacy, University of Pittsburgh Medical Center, Pittsburgh, Pennsylvania, USA; Department of Pharmacy, University of Pittsburgh Medical Center, Pittsburgh, Pennsylvania, USA; Department of Pharmacy, University of Kentucky Medical Center, Lexington, Kentucky USA; Department of Pharmacy, University of North Carolina Medical Center, Chapel Hill, North Carolina, USA; Division of Infectious Diseases, University of North Carolina at Chapel Hill School of Medicine, Chapel Hill, North Carolina USA

**Keywords:** endocarditis, injection drug use, serratia, gram negative, bacteremia

## Abstract

**Background:**

Infective endocarditis due to *Serratia* spp. (S-IE) has historically been considered a rare entity. Typically linked to injection drug use (IDU), S-IE appears to be a growing problem as the harms of unsafe drug use proliferate. However, guidance on therapy for Gram-negative IE remains limited.

**Methods:**

This was a retrospective analysis of adults treated for S-IE at 4 academic health systems in different US states from 2015 to 2021. Multivariable logistic regression analyzed the association of inpatient mortality with procedural management and combination antibiotic treatment.

**Results:**

A total of 159 cases of S-IE were identified with a qualitative overall increase across the period, and a peak in 2019, although trends varied by site. Seventy-five were due to IDU, 57% involved a single left-sided valve, and inpatient mortality was 21%. In adjusted analyses, including 117 cases from 3 sites, lower inpatient mortality was associated with procedural intervention (odds ratio 0.14; 95% confidence interval, .03–.64) and combination antibiotic therapy (odds ratio 0.15; 95% confidence interval, .03–.74).

**Discussion:**

In this multicenter study, we found that S-IE may be increasing, is commonly associated with IDU, is treated with varying strategies and carries high inpatient mortality. Procedural intervention and combination antibiotics were associated with lower mortality. Our study is limited by varying methods of case identification and a lack of data on clinical severity and surgical indications. Further study is urgently needed to define best management practices.

## Teaser

*Serratia* spp. infective endocarditis is described as a rare entity associated with injection drug use. However, in a cohort of 4 US health systems from 2015–2021, *Serratia* spp. infective endocarditis increased markedly over time and was associated with high inpatient mortality.

Infective endocarditis (IE) due to *Serratia* spp. (S-IE) has historically been considered rare, limited to case series, and most commonly described among people who inject drugs (PWID) [1, 2] However, the number of reports and case series on S-IE published over the past decade has increased, following surging rates of injection drug use (IDU) and opioid overdoses [3–6]. In a study of non-*Haemophilus*, *Aggregatibacter*, *Cardiobacterium*, *Eikenella*, and *Kingella* (HACEK) Gram-negative IE from 2011 to 2019 at 1 center in Tennessee, 43 cases were identified, with 20% with *Serratia marcescens* and 93% overall associated with a past or current history of IDU [7]. Another report of non-HACEK Gram-negative IE across a large health system in Pennsylvania from 2010 to 2021 revealed an emergence of *Serratia* spp. as the most prevalent organism in later years, particularly among PWID [8].

Guidance on the treatment of non-HACEK Gram-negative IE, however, remains limited [9], with recommendations based off a single cohort of 49 patients [10]. Current guidelines recommend both cardiac surgery and prolonged combination therapy with a β-lactam and a fluoroquinolone or aminoglycoside. However, optimal management may vary by organism [8].

Given the rise in IDU-associated IE and mounting reports of S-IE from individual health systems, we sought to examine trends, management strategies, and outcomes of S-IE in a multicenter cohort of 4 academic health systems.

## METHODS

We conducted a retrospective study of individuals aged 18 years or older with S-IE at 4 health systems in different states of the United States, from 2014 to 2021. Sites were composed of academic health centers or systems located in North Carolina, Pennsylvania, West Virginia, and Kentucky with wide catchment areas including urban, suburban, and rural regions. Two sites included data from the entire health system, whereas the other 2 were from the single tertiary academic centers. At 2 health systems (sites 1 and 3), cases of S-IE were identified by searching free text of the electronic medical record for the terms “Serratia” and “endocarditis.” The 2 other systems (sites 2 and 4) identified cases via review of positive blood cultures identified by the microbiology laboratory. Investigators at each site reviewed medical records to confirm that patients were documented to have positive cultures for *Serratia* spp. and were determined by treating clinicians to meet a diagnosis of infective endocarditis. Only valvular IE was included; patients with infections of cardiovascular implantable electronic devices only were not included. Data on demographics, risk factors, clinical presentation, management, and outcomes were abstracted from the medical records system at each site.

Three of 4 sites contributed data on antimicrobials. Combination treatment referred to the receipt of 2 or more antibiotics with *in vitro* activity against the *Serratia* spp. isolate for 72 hours or more. Antibiotics were grouped in the following mutually exclusive categories: piperacillin/tazobactam, third-generation cephalosporin, cefepime, carbapenem, fluoroquinolone, combination β-lactam/fluoroquinolone, combination β-lactam/aminoglycoside, other combination.

Descriptive statistics of the entire cohort were calculated. Trends in incidence of cases were examined overall and by site. Using bivariate logistic regression, we examined the association between inpatient mortality and site of care, age, sex, race, recent injection drug use history, prosthetic valve IE, prior IE, polymicrobial infection, operative management (defined as cardiac surgery or transcatheter aspiration vegectomy, TAV, to treat S-IE) and combination antibiotics with inpatient mortality. Inpatient mortality was defined as either inpatient death or discharge to hospice. Multivariable logistic regression assessed the association between inpatient mortality and either operative management or combination antibiotics, adjusting for factors significant in the bivariate models at *P* < .05. Analyses were performed with SAS v9.4 (Cary, NC). Trends were restricted to 2015–2021 because only 2 sites contributed cases from 2014. Other analyses included data from 2014.

The study was reviewed and approved by institutional review boards at each site. Data were collated and analyzed at the University of North Carolina.

## RESULTS

**Figure 1.**
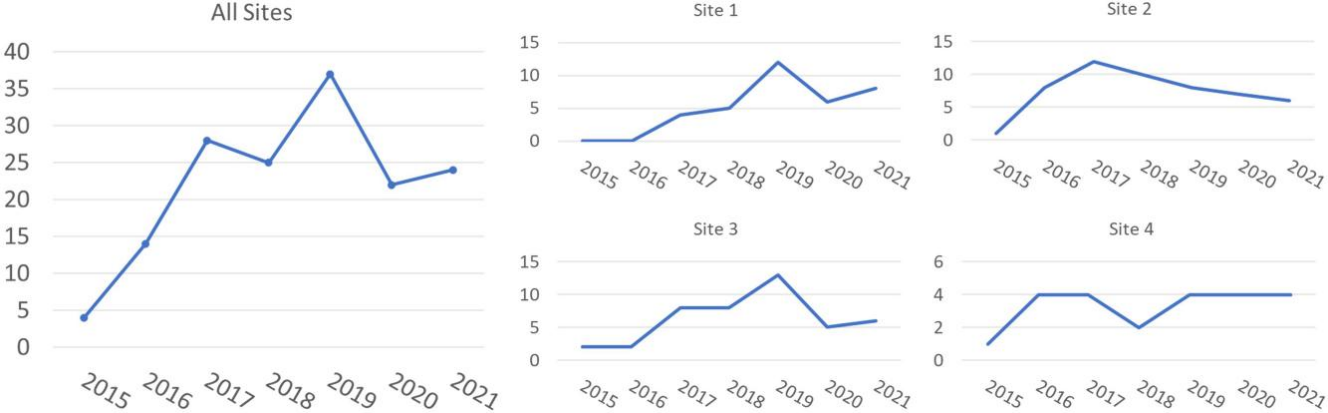
Trends in infective endocarditis due to *Serratia spp*., 2015–2021.

A total of 159 cases of S-IE were identified. Visually, yearly incidence qualitatively increased across the overall study period of 2015 through 2021 with a peak in 2019 (Figure 1); however, there was no linear trend in year-to-year incidence, based on linear regression (*P* = .13). Sites 1 and 3 had similar qualitative trends in incidence with steady increases over the study period, whereas sites 2 and 4 increased then plateaued around 2017. Most cases (Table 1) were among young people (106/159, 67%; aged 18–40 years), associated with IDU (120/159, 75%), and involved native valves (110/159, 69%). More than 50% of cases involved a single left-sided valve with an additional 8% involving multiple valves, which could include a left-sided valve. Of the 117 cases with species-level data, 116 were *S marcescens*; the 1 remaining case was classified as “other” but not specified. Inpatient mortality was 21%.

**Table 1.** Demographics, Clinical Characteristics, and Outcomes

	N = 159	Percent		N = 159	Percent
Age			Polymicrobial		
18–30	38	24%	No	90	60
31–40	68	43%	Yes	59	40
41–50	27	17%	Missing	10	
>50	26	16%	Valve		
Sex			Single right-sided valve	55	35
Male	89	56%	Single left-sided valve	89	57
Female	70	44%	Multiple valves	13	8
Race			Missing	2	
White	145	91%	Prosthetic valve IE		
Black	12	8%	No	110	69
Other	1	1%	Yes	49	31
Unknown	1	1%	Patient-directed discharge		
Risk factor			No	149	94
IDU	120	75%	Yes	10	6
Other	39	25%	Inpatient death or discharge to hospice		
Prior infective endocarditis			No	125	79
No	94	59%	Yes	34	21
Yes	65	41%	Readmission in 30 d		
			No	81	83
			Yes	17	17
			Unknown	61	

Abbreviation: IDU, injection drug use.

Of the 159 cases (Table 2), 57 (36%) had valve surgery and 3 had TAV. Data from the 3 sites with data on antimicrobials (N = 117) showed that most cases (n = 61, 57%) were treated with single-agent regimens. Antibiotic choices varied widely. Most regimens included single-agent treatment with a third-generation cephalosporin (9%), cefepime (26%) or carbapenem (14%), or combination treatment with a β-lactam backbone and a fluoroquinolone (23%) or an aminoglycoside (19%).

**Table 2.** Surgical and Antibiotic Management

	N = 159	Percent
Surgery		
No	102	64%
Yes	57	36%
Transcatheter aspiration vegectomy		
No	108	97%
Yes	3	3%
Unknown	48	
	n = 117	Percent
Combination antibiotic		
No	61	57%
Yes	46	43%
Treatment regimen		
Piperacillin/tazobactam	3	3%
Third-generation cephalosporin	10	9%
Cefepime	28	26%
Carbapenem	15	14%
Fluoroquinolone	5	5%
β-lactam and fluoroquinolone	25	23%
β-lactam and aminoglycoside	20	19%
Other combination	1	1%

Logistic regression revealed significantly higher odds of mortality with single left-sided valvular disease (unadjusted odds ratio [OR] 4.13; 95% confidence interval [CI], 1.48–11.52) as well as with site 1 (unadjusted OR 8.26; 95% CI, 1.77–38.49) and site 2 (unadjusted OR 6.00; 95% CI, 1.09–32.94) (Table 3). Significantly lower odds of mortality were associated with procedural intervention (surgery or TAV; adjusted OR 0.14; 95% CI, .03–.64) as well as combination antibiotics (adjusted OR 0.15; 95% CI, .03–.74).

**Table 3.** Multivariate Analysis Assessing for Odds of Mortality

	Unadjusted OR		
	Estimate	95% CI Low	95% CI High
Site			
1	1.00		
2	8.26	1.77	38.49
3	3.92	0.79	19.43
4	6.00	1.09	32.94
Age			
18–30	60.00	0.19	1.19
31–40	0.43	0.15	1.25
41–50	0.79	0.24	2.61
>50	1.00		
Sex			
Male	1.00		
Female	0.86	0.40	1.86
Race			
White	1.00		
Not White	1.00	0.26	3.82
Injection drug use			
No	1.00		
Yes	0.60	0.26	1.39
Valve			
Single right-sided valve	1.00		
Single left-sided valve	4.13	1.48	11.52
Multiple valves	3.00	0.62	14.62
Procedural intervention (surgery or TAV)			
No	1.00		
Yes	0.12	0.03	0.40
Combination antimicrobial treatment^a^			
No	1.00		
Yes	0.14	0.03	0.65

	Adjusted OR		
	Estimate	95% CI low	95% CI high
Procedural intervention (surgery or TAV)^a^			
No	1.00		
Yes	0.14	0.03	0.64
Combination antimicrobial treatment^a^			
No	1.00		
Yes	0.15	0.03	0.74

Abbreviations: CI, confidence interval; OR, odds ratio; TAV, transcatheter aspiration vegectomy.

^a^Site 4 excluded from analysis.

## DISCUSSION

Our study describes the largest cohort of S-IE cases to date. We found that S-IE cases increased from 2015 through 2021 across 4 different health systems, with wide geographic spread and variable trajectories by site. S-IE was associated with high inpatient mortality, despite primarily impacting young persons. Surgical intervention and combination therapy were each associated with significant lower odds of mortality.

Historic data describing *Serratia* endocarditis as rare is not reflective of current trends. One of the largest studies of non-HACEK Gram-negative IE published in 2007 described *Serratia* as an uncommon IE pathogen, even among PWID [10]. However, that cohort included limited US representation, and the prevalence of IDU has increased substantially since 2010 [11]. An analysis of the National Readmissions Database [12] found the proportion of IDU-related IE increased from 15.3% to 29.1% among all US cases of IE cases from 2010 to 2015. Given that IDU is the predominant risk factor for S-IE, it is not surprising that only in recent years has a surge in S-IE been observed.

Recent retrospective studies of *Serratia* bloodstream infections (BSI) have shown that a significant proportion of BSI may result in IE (12–30%), particularly among PWID [8, 13–16]. For example, McCann et al [14] retrospectively examined 103 patients with *Serratia* BSI at 4 community hospitals in Ohio from 2014 to 2018. Of the 103 patients, 42% were related to IDU and IDU-related cases experienced significantly more complications than non–IDU-related cases including IE, osteomyelitis, septic emboli, or epidural abscesses (52% vs 8%). The most common complications among all patients were IE (12%) and osteomyelitis (10%). In non-IDU cases, central line infections constituted the most common cause of *Serratia* BSI. Another single-center retrospective cohort study [15] of 67 *S marcescens* BSI from 2013 to 2019 in Maine found that 21% were due to IDU; endocarditis was identified solely among IDU-related infections (48% vs 0%), although PWID more often had echocardiography performed compared to non-PWID. All-cause mortality of S-IE at 12-months was 21%, which is consistent with the high inpatient mortality in our current study.

In a more recent analysis of 123 cases of Gram-negative IE from 2010 to 2021 [8] *Serratia* spp. were the most common species (43%) overall and were associated with vegetations >1 cm more frequently than other organisms (77% vs 47%). Although 52% of the 123 cases of Gram-negative IE were among PWID, 70% of the cases of S-IE were among PWID, suggesting that *Serratia* spp. have a particularly strong association with IDU. Furthermore, in 1 prospective study [17] of cardiac device infections in bacteremic patients found *S marcescens* posed a similar risk of causing prosthetic infections as *Staphylococcus aureus* despite constituting <10% of Gram-negative rod BSI. This study prompted the addition of *S marcescens* to the 2023 Duke Criteria as a typical organism for prosthetic valve IE [18].

The current thought that S-IE is a rare entity and not a typical organism for native valve IE by diagnostic criteria [18] may contribute to delayed or missed diagnosis and possibly account for the high associated mortality. Until 2023, the modified Duke criteria did not list any *Serratia* species as typical organisms with the updated 2023 criteria identifying *Serratia marcescens* as a typical organism only for those with prosthetic material [18]. Our study found 69% of S-IE cases involved native valves with 57% involving a single left-side valve and 8% involving multiple valves. Since transthoracic echocardiography is not as sensitive as transesophageal echocardiography for endocarditis [19], even if patients undergo cardiac imaging, cases may be missed if transesophageal echocardiography is not pursued. This may also be limiting evaluation of treatment approaches if certain cases are missed or only identified late, as delayed diagnosis would likely raise mortality.

It remains unclear why *Serratia* IE and associated poor outcomes occur predominantly among PWID. It has been suggested that perhaps tap water used to dissolve drugs may be a source [14], but to our knowledge no formal research has confirmed this or explained why *Serratia* spp. has emerged as the predominant waterborne pathogen. Other theories include susceptibility to IE from higher inoculum related to direct injection into the bloodstream as well as valvular destruction predisposing to IE related to IDU and prior episodes of IE, although our study found the majority (59%) of patients with S-IE had not had a prior episode of IE. Another potential explanation is drug use equipment itself acting as a vector for bacterial disease transmission and perhaps selecting for more virulent, hardy biofilm-producing organisms. One recent study [20] cultured bacteria from discarded syringes collected by harm reduction and outreach organizations. Of 160 syringes, 59% had culturable organisms including *S marcescens*. This study suggests that bacteria can colonize syringes with the potential to be transmitted if syringes are shared or reused. More research is needed so that appropriate harm reduction strategies can be devised and PWID can be educated on how to reduce their risk of such infections.

Current endocarditis treatment guidelines state that use of combination therapy with a β-lactam plus a fluoroquinolone or aminoglycoside is “reasonable” for non-HACEK Gram-negative (not specific to *S marcescens*) endocarditis [9]. Although this guidance does not come from definitive trials, it may be influencing treatment approaches among infectious diseases providers [16]. Although we were unable to conclusively compare mortality outcomes between combination therapy with fluoroquinolones versus aminoglycosides because of small sample sizes, others have found reduced mortality with fluoroquinolone combination therapy in Gram-negative endocarditis [21]. This may be explained by the inability of positively charged aminoglycosides to effectively permeate the negatively charged exopolysaccharide in biofilm infections [22], whereas fluoroquinolones may be more effective in permeating biofilms. This distinction may be important because biofilm formation is of particular concern in S-IE. Despite mortality benefits observed with combination therapy in our study, such combination may put patients at risk for adverse effects in particular from aminoglycosides [13].

Although our study constitutes the largest S-IE cohort to date, there are several limitations to our analysis. First, this was a retrospective investigation. Second, sites utilized different strategies for to retrospectively identify S-IE cases at their institutions. This almost certainly leads to underestimation in incidence varying by site and may explain the variation in trends and outcomes seen across sites. Third, we were unable to collect data on the extent or severity of infection, and why certain treatments including surgical intervention were pursued or not. Our study identified cases across a variety of hospital systems, however, which encompassed a wide array of practices and approaches in different regions of the United States.

In conclusion, further study of non-HACEK Gram-negative IE, specifically due to *Serratia* spp., is needed to clarify optimal therapeutic management, especially in midst of rising rates of IDU. Further research on the microbiologic propensity of *Serratia* spp., particularly *S marcescens*, to cause IE and other invasive infections is needed to support accurate risk assessment and inform potential harm reduction strategies. The incidence of S-IE may be low for prospective trials, but larger cohorts could potentially identify enough cases to make meaningful recommendations on management strategies. *Serratia* bloodstream infection and IE should not be underestimated, particularly among PWID.

## Notes

***Patient consent statement***. Our study did not include factors necessitating patient consent. The study was reviewed and approved by institutional review boards at each site.

***Financial support***. A.J.S. is funded by National Institute on Drug Abuse (K23DA049946).
